# One-step surgery for acute ischemia of the jejunal loop after pancreatoduodenectomy: report of a case

**DOI:** 10.1186/s40792-016-0153-6

**Published:** 2016-03-14

**Authors:** Hiroto Kayashima, Takashi Maeda, Noboru Harada, Takanobu Masuda, Takahiro Ohmine, Shohei Yamaguchi, Ayumi Matsuyama, Motoharu Hamatake, Shinichi Tsutsui, Hiroyuki Matsuda

**Affiliations:** Department of Surgery, Hiroshima Red Cross Hospital and Atomic Bomb Survivors Hospital, 1-9-6 Senda-machi, Naka-ku, Hiroshima 730-8619 Japan

**Keywords:** Whipple procedure, Complication, Thrombosis

## Abstract

**Background:**

Pancreatoduodenectomy (PD) is an extensive surgery, and its complications are grave. Acute ischemia of the jejunal loop due to thrombosis of the superior mesenteric vein (SMV) and its branches is one of the most dangerous complications that, although rare, if left untreated leads to abdominal sepsis and death of a patient.

**Case presentation:**

A 77-year-old man underwent PD for pancreatic cancer. On postoperative day 2, the patient developed a severe anemia with hypotension. The computed tomography showed acute ischemia of the jejunal loop due to thrombosis. The emergent surgery was performed. The removal of the ischemic intestine and re-anastomoses of the biliary and pancreatic ducts could be performed all at once because necrosis and inflammation were still very mild in early stage.

**Conclusion:**

If suspicion for thrombosis of the SMV and its branches is raised, re-laparotomy should be considered. Early re-operation can lead to removal of the ischemic intestine and re-anastomoses in one-step surgery.

## Background

Pancreatic cancer is one of the most lethal malignancies and is associated with a very poor overall survival, and surgery remains the only option for the possibility of cure for patients with localized pancreatic cancer [1]. Pancreatoduodenectomy (PD) is indicated for pancreatic head cancer. As surgical techniques and chemoradiation regimens have evolved, the indication has been expanded. As a result, superior mesenteric vein (SMV) and portal vein (PV) reconstructions and venous interposition grafts have become quite common [2, 3].

The most common complications of PD can be divided into four groups: postoperative seromas and abscesses, delayed gastric emptying, hemorrhage (early vascular bleeding), and anastomotic leakage [4]. Nevertheless, other complications, much less frequent but life-threatening, exist. One of such complications is thrombosis of the SMV and its branches. Clinical symptoms are untypical, obscure, and characterized by slow progress, all of these covered by early postoperative period [5, 6]. The conservative therapy, such as administration of heparin or thrombolytic agents, does not differ from that of the thrombosis in any other localization. However, if the conservative therapy was ineffective, mortality of the patients with necrotic intestine and with developed abdominal sepsis is extremely high. Prognosis of the patient depends on the clinical state, early identification of this complication, and aggressive treatment [5–7].

We herein report a case of a male patient who had been diagnosed with acute ischemia of the jejunal loop due to thrombosis of the SMV branches 2 days after PD for pancreatic cancer and was performed the removal of the ischemic intestine and re-anastomoses of the biliary and pancreatic ducts in one-step surgery because necrosis and inflammation were still very mild in early stage.

## Case presentation

The patient was a 77-year-old man who was diagnosed with pancreatic head cancer with asymptomatic worsening of diabetes mellitus and admitted to our department for elective surgery. The patient’s past history had no obvious problems. Subtotal stomach-preserving PD with lymph node dissection was performed. There was no tumor involvement of the PV and SMV; therefore, no venous reconstruction was performed. Reconstruction was performed by the Roux-en-Y method. The jejunal loop was pulled out through the transverse mesocolon, and then pancreaticojejunostomy and hepaticojejunostomy were performed by retrocolic anastomoses. The pancreatic duct was anastomosed to a small opening on the jejunal wall using eight interrupted 6-0 monofilament absorbable sutures. The pancreatic tube was inserted and secured at the posterior wall of the anastomosis as an external drainage. The biliary duct was anastomosed to the jejunum using 16 interrupted 5-0 monofilament absorbable sutures with a lost tube. Gastrojejunostomy was performed using linear stapling devices by an antecolic anastomosis, and then Braun anastomosis was added. There was no abnormal finding on the reconstructed jejunal loop intraoperatively. After the surgery, the patient was admitted to the intensive care unit under stable condition.

**Fig. 1:**
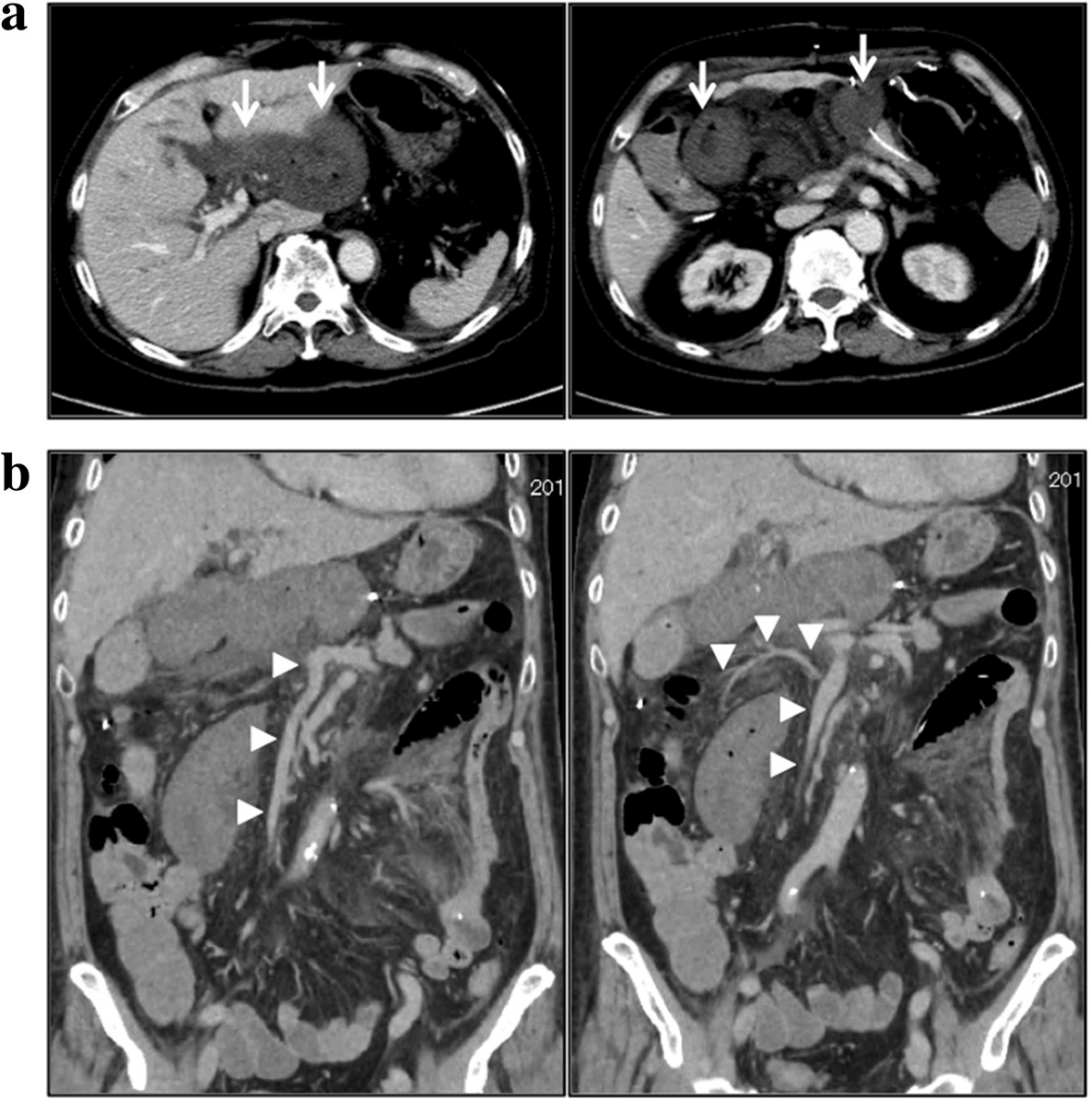
The findings of enhanced CT on POD 2: **a** Axial images showed acute ischemia of the jejunal loop with a thickened wall (*white arrows*). Remnant pancreas were normal. **b** Coronal images showed that there was no obvious thrombosis of the SMV and its branches (*white arrowheads*)

On postoperative day (POD) 1, there was a little amount of bleeding from the nasogastric tube. However, no signs of elevated levels of amylase in exudate fluid from the drains and no clinical abdominal tenderness with peritoneal signs were found. On POD 2, the abdomen was without peritoneal signs, audibly with peristaltic movement, and the drains showed no signs of pancreatic fistula and intraabdominal bleeding. However, the patient developed a severe anemia with hypotension. The enhanced abdominal computed tomography (CT) showed acute ischemia of the jejunal loop with a thickened wall (Fig. 1a). Although there was no obvious thrombosis of the SMV and its branches (Fig. 1b), D-dimer was elevated up to 7.0 μg/mL (normal range < 1.0 μg/mL) in the serum. A venostatic jejunal loop from venous thrombosis was highly suspected clinically; therefore, the emergent surgery was performed.

**Fig. 2:**
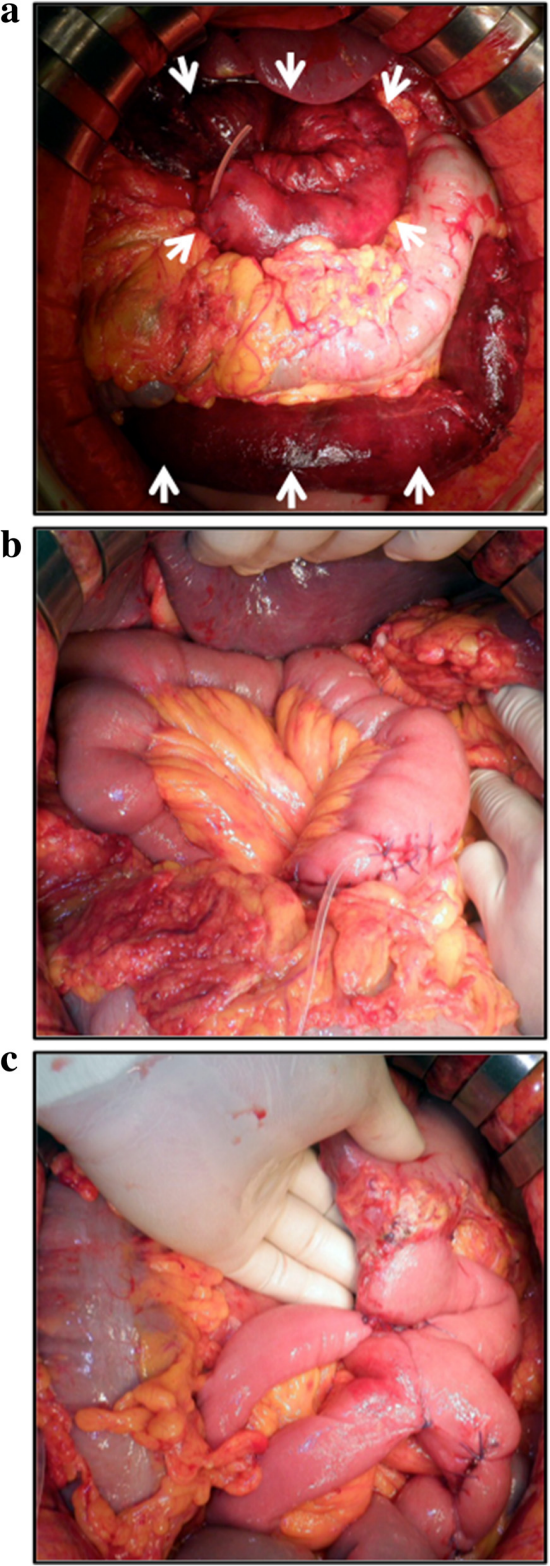
The intraoperative findings of emergent surgery: **a** The reconstructed jejunal loop became congested severely and appeared to be ischemic but not necrotic (*white arrows*). **b** The degrees of inflammation around pancreaticojejunostomy and hepaticojejunostomy were still very mild, and there were no signs of pancreatic fistula and bile leakage. Therefore, we performed the removal of the ischemic intestine and re-anastomoses of the biliary and pancreatic ducts all at once. **c** The gastrojejunostomy had no problem and did not need to be re-anastomosed

Intraoperative findings showed severe venous congestion of the jejunal loop. The intestine appeared to be ischemic but not necrotic (Fig. 2a). However, the intestine around the site of Braun anastomosis reconstructed in the first operation was clearly viable. There were no any compression sites or injuring parts of the jejunal loop. The degrees of inflammation around pancreaticojejunostomy and hepaticojejunostomy were still very mild, and there were no signs of pancreatic fistula and bile leakage. Therefore, we performed the removal of the intestine from the jejunal loop with acute ischemia to the site of Braun anastomosis reconstructed in the first operation and re-anastomoses of the biliary and pancreatic ducts all at once (Fig. 2b). The normal intestine was pulled out through the same site of the transverse mesocolon as the first operation, and then pancreaticojejunostomy and hepaticojejunostomy were performed by retrocolic anastomoses without any difficulty. The gastrojejunostomy had no problem and did not need to be re-anastomosed (Fig. 2c). At last, jejunojejunostomy was performed at the site of Braun anastomosis in the first operation. The histological findings of the resected intestine showed recent hemorrhagic infarct and multiple fresh venous thromboses in the wall and mesenterium. These results might suggest that the cause of this congestion was due to the thromboses of the SMV branches.

His postoperative course was uneventful except for delayed gastric emptying. The patient was released from the hospital under no anticoagulant therapy on POD 35 after the secondary operation.

### Discussion

As surgical and oncologic treatment for pancreatic cancer has been improved during recent years, the indication for PD has been expanded. In the past days, the only truly resectable tumors were those without involvement of the superior mesenteric artery (SMA), celiac artery, hepatic artery (HA), PV, and SMV. However, the past few years have seen the development of a new category of borderline resectable tumors, including tumors that involve <180° of the circumference of the SMA, abut or encase the HA for a short segment, or narrow (or occlude) the PV or SMV for a short segment with surgical options for reconstruction [1, 2]. As a result, the complexity and incidence of surgical venous reconstructions have markedly increased, and it is not rare for patients to undergo venous resections with either primary anastomosis or the insertion of a venous interposition graft. Accordingly, it is not surprising that there is a significant incidence of venous thrombosis (PV or SMV) after PD. It has been reported that 17 % of patients developed venous thrombosis [8] and the incidence may be significantly higher for more complex reconstructions. The mesenteric venous thrombosis (MVT) impairs venous return from the bowel, resulting in venous engorgement and ischemia. With rapid and complete occlusion of mesenteric veins, there may be insufficient time for development of a collateral circulation leading to transmural bowel infarction. The transition from normal to ischemic bowel is gradual, unlike in arterial ischemia when it is more abrupt. Arterial spasm secondary to venous engorgement may occur, with resulting irreversible bowel ischemia [9]. The development of the MVT can have disastrous consequences, including intestinal ischemia, uncontrolled ascites, hepatic ischemia, and death [10].

However, identification of the thrombosis of the SMV and its branches after PD in early postoperative course is of great difficulty by fact that clinical symptoms are non-specific and covered by postoperative paralysis of the gastrointestinal tract and modified pain reaction by the administration of the analgesic medications. Furthermore, specific biomarkers for intestinal ischemia have yet to be determined. It has been reported that D-dimer is in part useful to assess acute thromboembolic occlusion of the SMV; however, the use of D-dimer as a marker for ischemia has high sensitivity, but low specificity, and could be used for exclusion of diagnosis at best [5, 11].

Treatment of thrombosis of the SMV and its branches is divided into conservative, interventional, and surgical treatments. The initial treatment goal is cessation of thrombotisation and enabling of the body’s fibrinolytic activity for destruction of the thrombus. Administration of the therapeutic dosage of heparin provides immediate effect. Another possibility is thrombolysis administrated locally using interventional therapy [5–7]. However, if there is the slightest doubt about acute bowel ischemia, surgical revision should be considered. Surgical therapy has two goals. The first goal is facilitation of venous flow, and the second one is assessment of the vitality of the bowel with resection of necrotic segments. In contrast to arterial ischemia, the border between ischemic and livid bowel is less visible and identifiable, and second-look laparotomy should be indicated if in doubt [12].

Although there are a few reports of acute SMV thrombosis after PD, they are composed of either case reports or very small series of patients with heterogeneous clinical conditions. Therefore, the true cause of acute SMV thrombosis remains unknown [10]. In this case, although there were no obvious compression sites or injuring part of the jejunal loop in the secondary operation, mechanical injury to SMV branches could be considered as the main cause of thrombosis of SMV branches in early postoperative period. Leakage of pancreatic juice or bile could cause thromboses of the PV or SMV; however, there were no signs of pancreatic fistula and bile leakage. Usually, it is difficult to evaluate the accurate extent of the ischemic area in emergency situations like mesenteric ischemia. Although some studies have reported the usefulness of laser fluorescence angiography, confocal laser endomicroscopy, or contrast-enhanced ultrasonography for a real-time assessment of bowel viability [13–15], these assessments need specialized equipments and we cannot use such techniques commonly. Indeed, we did not use such tools in this case. In this case, clinical estimation of the viability of the congestion area was relatively easy and we removed the intestine from the jejunal loop with acute ischemia to the site of Braun anastomosis reconstructed in the first operation. The intestine around Braun anastomosis was clearly viable. Furthermore, the degrees of inflammation around pancreaticojejunostomy and hepaticojejunostomy were still very mild; therefore, we could perform the removal of the ischemic intestine and re-anastomoses of the biliary and pancreatic ducts all at once. If the ischemic area of the intestine was ambiguous, or if the degrees of inflammation around pancreaticojejunostomy and hepaticojejunostomy were severe, we might perform the external fistulations of the biliary and pancreatic ducts and second-look operation within the next day or so.

## Conclusions

After PD, early postoperative thrombosis of the SMV and its branches is a real and potentially catastrophic complication leading to acute bowel ischemia. Awareness of the potential for acute ischemia of the jejunal loop due to thrombosis of the SMV and its branches will allow for early detection and one-step surgery with the removal of the ischemic intestine and re-anastomoses of the biliary and pancreatic ducts all at once because necrosis and inflammation were still very mild in early stage.

## Consent

Written informed consent was obtained from the patient for publication of this Case Report and any accompanying images. A copy of the written consent is available for review by the Editor-in-Chief of this journal.

## Abbreviations

CT: computed tomography
HA: hepatic artery
MVT: mesenteric venous thrombosis
PD: pancreatoduodenectomy
POD: postoperative day
PV: portal vein
SMA: superior mesenteric artery
SMV: superior mesenteric vein
